# Structure Assignment of Seized Products Containing Cathinone Derivatives Using High Resolution Analytical Techniques

**DOI:** 10.3390/metabo11030144

**Published:** 2021-02-27

**Authors:** João L. Gonçalves, Vera L. Alves, Joselin Aguiar, Maria J. Caldeira, Helena M. Teixeira, José S. Câmara

**Affiliations:** 1 CQM—Centro de Química da Madeira, Campus Universitário da Penteada, Universidade da Madeira, 9020-105 Funchal, Portugal; vera.alves@staff.uma.pt (V.L.A.); joselin.aguiar@staff.uma.pt (J.A.); 2 Laboratório de Polícia Científica da Polícia Judiciária, Novo edifício-sede da Polícia Judiciária, Rua Gomes Freire, 1169-007 Lisboa, Portugal; joao.caldeira@pj.pt; 3 Instituto Nacional de Medicina Legal e Ciências Forenses, I.P., Polo das Ciências de Saúde (Polo III), Azinhaga de Santa Comba, 3000-548 Coimbra, Portugal; helena.m.teixeira@inmlcf.mj.pt; 4 Faculdade de Medicina da Universidade de Coimbra, Azinhaga de Santa Comba, Celas, 3000-548 Coimbra, Portugal; 5 Faculdade de Ciências Exatas e da Engenharia, Campus da Penteada, Universidade da Madeira, 9020-105 Funchal, Portugal

**Keywords:** chemical characterization, new psychoactive substances, synthetic cathinones, FTIR, GC-MS, NMR

## Abstract

The innovation of the new psychoactive substances (NPS) market requires the rapid identification of new substances that can be a risk to public health, in order to reduce the damage from their use. Twelve seized products suspected to contain illicit substances were analyzed by attenuated total reflectance Fourier transform infrared spectroscopy (ATR-FTIR), gas chromatography coupled to mass spectrometry (GC-MS), and nuclear magnetic resonance spectroscopy (NMR). Synthetic cathinones (SCat) were found in all products, either as a single component or in mixtures. Infrared spectra of all products were consistent with the molecular structure of SCat, showing an intense absorption band at 1700–1674 cm^−1^, corresponding to the carbonyl stretching, a medium/strong peak at 1605–1580 cm^−1^, indicating stretching vibrations in the aromatic ring (C=C) and bands with relative low intensity at frequencies near 2700–2400 cm^−1^, corresponding to an amine salt. It was possible to identify a total of eight cathinone derivatives by GC-MS and NMR analysis: 4′-methyl-α-pyrrolidinohexanophenone (MPHP), α-pyrrolidinohexanophenone (α-PHP), 3-fluoromethcathinone (3-FMC), methedrone, methylone, buphedrone, *N*-ethylcathinone, and pentedrone. Among the adulterants found in these samples, caffeine was the most frequently detected substance, followed by ethylphenidate. These results highlight the prevalence of SCat in seized materials of the Portuguese market. Reference standards are usually required for confirmation, but when reference materials are not available, the combination of complementary techniques is fundamental for a rapid and an unequivocal identification of such substances.

## 1. Introduction

In the last two decades, a tremendous change in the illicit drug market has become evident, with the emergence of a “new generation” of psychoactive substances [1]. Mimicking the effects of illicit drugs, and sometimes being traded alongside them, these NPS have introduced many challenges to legal systems, as well as emergency and drug treatment services [2]. Among these substances, SCat rapidly gained popularity, especially among young people, and currently constitute the second largest group of NPS monitored in Europe, with almost 140 identified substances so far [3,4].

**Figure 1:**
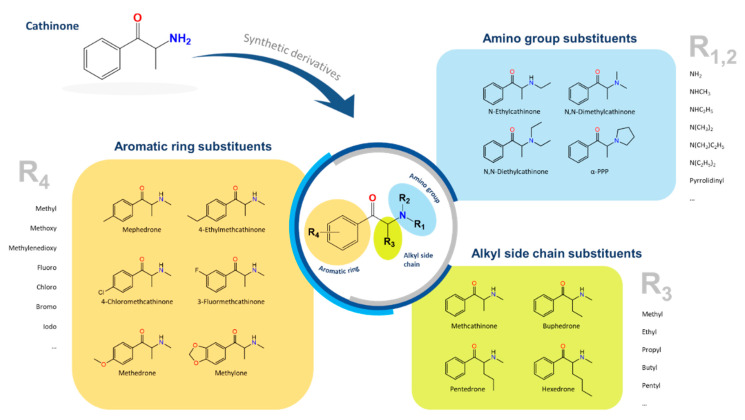
General chemical structure of SCat and some representative examples of functional group substituents on the aromatic ring, the alkyl side chain and the amino group.

Natural cathinone, which is the major active component of the Khat plant (*Catha edulis* Forsk), has been used as a prototype for the development of numerous synthetic derivatives [5,6]. In general, functional group substitutions in the cathinone occur in three distinct regions of the molecule, namely the amino group, the alkyl side chain, and the aromatic ring (Figure 1), which has led to a multitude of derivatives to the street and cyber drug markets [7,8].

SCat can be found on the market in products sold as “bath salts” or “plant feeders”, in different forms, including tablets, capsules, crystals or powders [1,9]. These substances are usually taken orally, either by swallowing capsules or ‘bombing’, where the powder is wrapped in a cigarette paper and subsequently swallowed [10,11]. Inhalation is another common mode of administration of these substances, whereas gingival delivery, intravenous or intramuscular injection and rectal administration are less common routes [11,12].

Similarly to other stimulant drugs like cocaine or 3,4-methylenedioxymethamphetamine (MDMA), SCat interact with monoamine membrane transporters, leading to an increase in serotonin concentrations (5-HT), dopamine (DA) and noradrenaline (NA) [8,10,13]. In general, these substances create a feeling of euphoria, alertness, empathy, and increased libido, being highly sought after as an alternative to traditional drugs of abuse [14,15]. However, the consumption of SCat can lead to a varied and unpredictable number of side effects, and in many cases, are linked to aggression and violent behavior [16,17]. Despite their relatively short time on the clandestine market, SCat have been responsible for several cases of acute poisonings and fatal overdoses [18,19,20,21]. Acute intoxication with SCat can produce a wide range of symptoms, including psychiatric disturbances (panic attacks, hallucinations, paranoid psychosis, and suicidal ideations), sympathomimetic toxidrome (agitation, increased aggression, hypertension, tachycardia, chest pain, cardiac arrest), seizures, acid-base imbalance and multi-organ failure [1,4,22,23]. In general, the most frequent cause of intoxication and fatalities is related to the unknowledge about the product content and its purity, or even with the concentration of the psychoactive substance(s) in the administered dose [24].

Up to now, many efforts and some progress has been made to control the sale and distribution of these substances. From temporary banning orders to consumer legislation, governments are using a host of tools in an attempt to curtail the free sale and distribution of these products [2]. In Portugal, the commercialization of SCat has been outlawed since 2013, after the introduction of the Decree-Law no. 54/2013, of 17 April, which defines the legal framework for the prevention and protection against the advertising and commerce of the NPS and forbids the production, importation, exportation, publicity, distribution, possession and selling of 159 NPS, including 34 SCat [25]. Despite measures that have helped to reduce the supply of NPS, by closing down the so-called “smartshops” (retail establishments specialized in selling psychoactive substances and related paraphernalia), SCat continue to appear in the drug market and its abuse still represents a public health issue.

The ever-evolving nature of these substances has created many analytical challenges for forensic and chemistry laboratories, due to the large number of potential compounds to be investigated, and for which there are no available reference standards [26,27]. 

Although it is possible to routinely analyze the already identified substances, using appropriate instrumentation, such as liquid or gas chromatography coupled to mass spectrometry (LC-MS and GC-MS), the identification of unknown substances generally requires a structural elucidation before being included in routine methods [28]. Typically, this process requires the use of NMR spectroscopy to identify the chemical structure of the compound. However, the structural similarities of some substances, such as the SCat, may offer analytical challenges, which make the analysis particularly difficult. In such situations, the selection of analytical techniques can be challenging, since each technique has its own advantages that may be more or less suitable, depending on the situations. This highlights the importance of using complementary analytical techniques to confirm the identity of a substance [28].

In this context, the present study describes and discusses the analytical assays performed to identify the components of 12 products seized in Portugal, suspected to contain SCat. Structure elucidation of compounds was carried out by ATR-FTIR, GC-MS and NMR spectroscopy.

## 2. Results and Discussion

The identification of products containing NPS is a challenging task, as the present substances do not generally correspond to the label, and can contain a wide range of impurities and/or adulterants [23]. The products were presented in brightly colored packaging or, occasionally, in transparent plastic bags with the trade name, the supposed composition, the product quantity along with the warning “not for human consumption”. Most products (83%) were in powder form, and only two products were presented in the crystal (product 2) and tablets (product 10) forms (Table 1). The predominant color of the powders and tablets were white, whereas the crystal had a brownish color. In relation to the composition indicated on the packaging, all had “ketones” in the list of ingredients, presumably indicative of the presence of Scat, except products 1 and 2. In addition, most powder products also referred the presence of caffeine and glucose. The composition indicated on the packaging of the tablets was more elaborated, with the information of the active principle and of other excipients related to the production of the tablets.

### 2.1. ATR-FTIR Analysis

The infrared spectra of all seized materials were consistent with the molecular structure of SCat (Figure S1 Supplementary Material). All spectra showed a prominent absorption band around 1700–1674 cm^−1^ that corresponds to the carbonyl group stretching (C=O).

**Figure 2:**
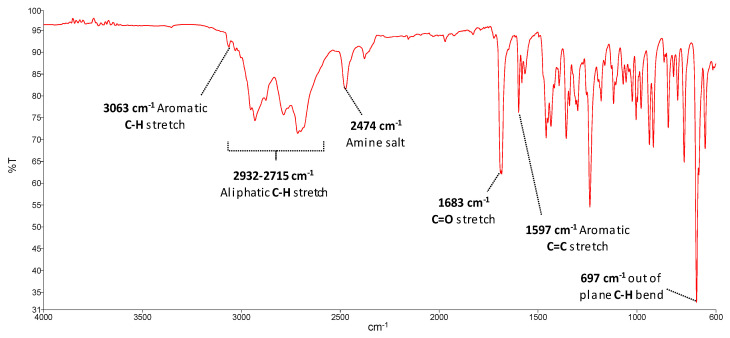
FTIR spectrum of product 8 (“Charlie”).

The intensity of this band exhibited variation among the samples, but was the major peak in many cases. Bands observed at 3063–3024 cm^−1^ and 2952–2711 cm^−1^ can be assigned to aryl C–H and alkyl C–H stretch vibration, respectively. On the other hand, the medium/strong band observed at 1605–1580 cm^−1^ can indicate stretch aromatic ring vibrations (C=C), while a set of bands in the range of 2700–2400 cm^−1^ can be assigned to the amine salt [28,29]. Figure 2 describes an example of the infrared bands identified in one seized product (product 8).

For absorptions below 1500 cm^−1^, the differences between samples were more significant. A strong band observed in products 9 and 10 at 1259 cm^−1^ dominates the IR spectrum and could indicate a C–O–C stretching [30,31]. In general, the out-of-plane C–H bending bands in the region of 675–900 cm^−1^ are used to differentiate between substituted aromatic compounds [32,33]. For monosubstituted benzene rings, out-of-plane C–H bending bands fall between 770–730 cm^−1^ and 710–690 cm^−1^ and are often the most intense bands in the spectra [32,34]. In this sense, products 2–8 and 12 showed a pattern consistent with these characteristics, indicating that we may be facing compounds with monosubstituted aromatic rings. It is important to note that "Bloom" products (products 3–7) had identical IR spectra, which may indicate that they have the same chemical composition.

### 2.2. GC-MS Analysis

**Figure 3:**
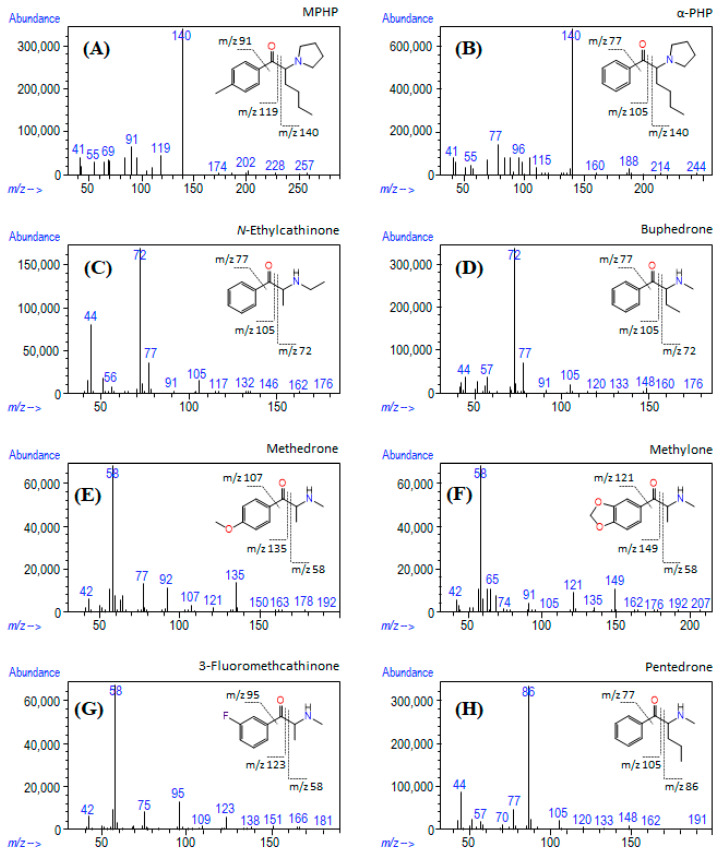
EI mass spectra of (**A**) MPHP, (**B**) α-PHP, (**C**) *N*-ethylcathinone, (**D**) buphedrone, (**E**) methedrone, (**F**) methylone, (**G**) 3-FMC and (**H**) pentedrone found in seized products.

Methanolic solutions of the seized materials were directly analyzed by GC-MS, resulting in different chromatographic profiles (Figure S2, Supplementary Information). The EI mass spectra for most compounds were consistent with the fragmentation pattern of SCat, showing the presence of iminium cations as the base peak and small or absent intensities of the molecular ions (Figure 3). Table 2 shows the identified compounds, their molecular formulas, retention time, the base peak and other characteristic ions for the identified compounds.

**Figure 4:**
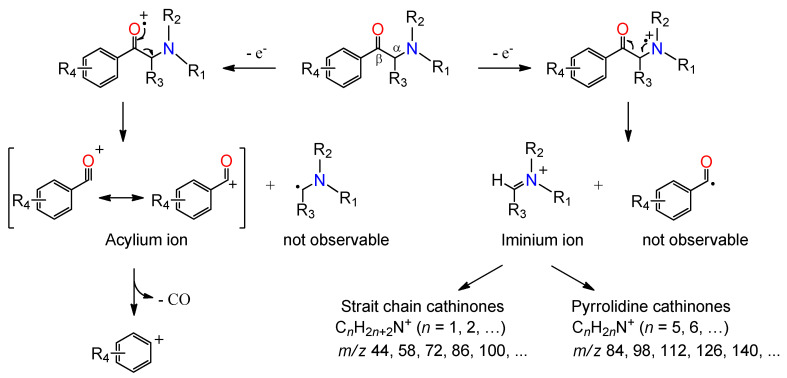
General mass spectra fragmentation pattern of SCat under EI conditions (adapted from Zuba [35]).

As reported by Zuba [35], the α-cleavage process is a key feature of the mass spectral fragmentation of SCat. This process results in the formation of the iminium ion by fragmentation of the C–C bond between the α and β carbon atoms under EI conditions (Figure 4). In the case of cathinones with a straight-chained aliphatic side ring, the structural formula of the base iminium ion is represented by C_n_H_2n+2_N^+^ (*n* = 1, 2, …), resulting in a base peak of mass-to-charge ratio (*m*/*z*) 44, 58, 72, 86, 100 and so on, depending on the number of carbon atoms contained in the iminium ion [35,36]. *N*-Ethylcathinone, buphedrone, pentedrone, methedrone, methylone and 3-FMC are SCat that produce typical C_n_H_2n+2_N^+^ iminium ions. For cathinones with a pyrrolidine ring in the side chain, such as MPHP and α-PHP, fragmentation leads to the formation of characteristic ions represented by the structural formula C_n_H_2n_N^+^ (*n* = 5, 6,…), which corresponds to R_3_CH=N^+^(C_4_H_8_) species [35]. The consequent fragmentation of the pyrrolidine ring leads to the formation of characteristic ions *m*/*z* 70, 55, 42 and 41 [35,37].

Another common reaction observed in SCat under EI condition is the formation of the acylium ion. In general, this process takes place at the same location as for the formation of the iminium ion and involves the dissociation of the C_α_–C_β_ bond, leaving behind an acylium ion [36]. The consequent loss of carbon monoxide (CO) from the acylium ion results in the formation of the phenyl cation, as indicated in Figure 4. The phenyl (*m*/*z* 77), methylphenyl (*m*/*z* 91), and fluorophenyl (*m*/*z* 95) cations are some representative examples of ions produced by the loss of carbon monoxide from the corresponding acylium ions at *m*/*z* 105 (benzoyl ion), 119 (methylbenzoyl ion) and 123 (fluorobenzoyl ion). Although these fragmentation patterns in the mass spectra provide structural information about the elemental composition, they do not differentiate structural isomers with different substitution patterns on the aromatic ring. 

Unfortunately, the EI method is often limited to differentiate structurally similar cathinones. The similar fragmentation patterns in some of these compounds, associated with the absence of molecular ion peak, may produce ambiguous mass spectra [38]. However, the differentiation of these compounds can be achieved using an alternative approach, such as chemical derivatization, which can represent a straightforward and cost-effective way to improve the capability of compound identification [39,40].

TFAA is one of the most widely used derivatizing agents, known to react with the primary and secondary amine groups of the amphetamine-type stimulants [41,42]. Derivatives are formed via acylation, where the amine hydrogen is removed and replaced with a trifluoroacyl group [43]. Not all substituted cathinones can be directly derivatized using TFAA, which requires, at least, one hydrogen atom in the amino group or an active hydrogen atom from other functional groups (e.g., –OH, –COOH).

**Figure 5:**
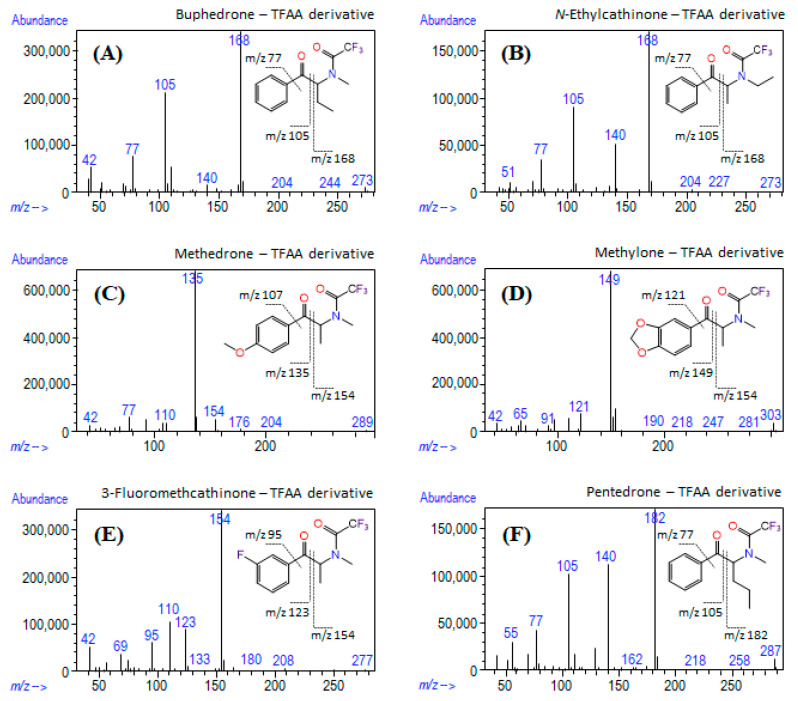
EI mass spectra of (**A**) buphedrone (**B**) *N*-ethylcathinone (**C**) methedrone (**D**) methylone (**E**) 3-FMC and (**F**) pentedrone after derivatization with TFAA.

After derivatization and knowing the molecular weight and ions resulting from fragmentation, it was possible to identify the chemical structures of the formed compounds. Figure S3 (Supplementary Information) shows the typical GC-MS chromatograms of the seized products after derivatization with TFAA, and Figure 5 describes the respective mass spectra of SCat after derivatization. Table 2 shows the identified TFAA derivative compounds, their molecular formulas, retention time, the base peak and other characteristic ions for the identified compounds.

**Figure 6:**
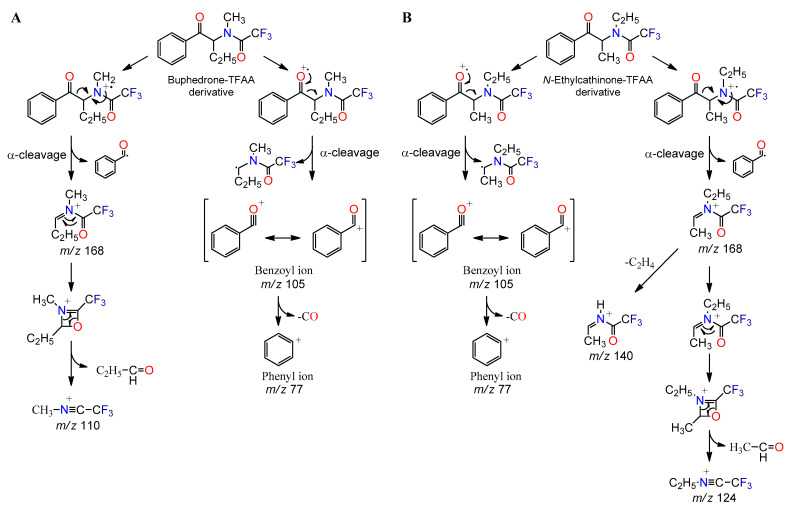
Probable fragmentation pathways of TFAA derivatives of, (**A**) buphedrone, and (**B**) *N*-ethylcathinone.

Derivatization of SCat with TFAA produced a better chromatographic resolution and increased mass spectral abundances for molecular and fragment ions. Before derivatization, products containing *N*-ethylcathinone and buphedrone (products 3–8) had a poor resolution, and the fragmentation patterns observed on the mass spectra of both compounds were very similar. After derivatization, these SCat showed better chromatographic separation, with larger, narrower, and more symmetric chromatographic peaks, and the mass fragmentation pattern of both compounds were readily distinguished by the different relative abundance of two common fragment ions (*m*/*z* 140 and 110). In general, SCat with a methyl substituent on the nitrogen atom, such as buphedrone, have a characteristic ion at *m*/*z* 110 ([CH_3_–N≡C–CF_3_]^+^). This cation may be formed from the decomposition reaction of the TFAA imine specie at *m*/*z* 168 (Figure 6A). *N*-Ethylcathinone with an ethyl substituent in the amino group produced analogous cation at *m*/*z* 124 corresponding to [C_2_H_5_–N≡C–CF_3_]^+^ (Figure 6B), with low relative abundance (2%). In addition, both compounds showed a characteristic ion at *m*/*z* 140. This cation is more abundant in *N*-ethylcathinone (29%) than in buphedrone (4%) and is probably originated from a rearrangement of the ethyl group of the *m*/*z* 168 cation to lose ethylene.

We can conclude, based on these results and considering the number of potential compounds to be investigated, associated to the lack of reference standards and the existence of a wide diversity of isomers with identical fragmentation patterns under EI conditions, that the derivatization with TFAA showed to be an effective strategy to confirm the identity of these substances, allowing us to obtain more specific structural information of these compounds. Thus, through the GC-MS analysis, it was possible to identify a total of 11 different substances, belonging, the most of them, to the class of SCat. MPHP was identified as the main compound in product 1, while α-PHP was the main component in product 2. Both substances showed characteristic mass spectral fragmentation pattern with SCat containing a pyrrolidine ring in the side chain. Methylone was identified as the main component in product 10, and together with caffeine in product 9. “Bloom” products (products 3–7) had a similar chromatographic profile and identical chemical composition, which means that they probably belong to the same batch. *N*-Ethylcathinone, buphedrone, methedrone, caffeine and ethylphenidate were the identified substances in these samples. It was also confirmed by GC–MS that products 8, 11 and 12 contained *N*-ethylcathinone, making this SCat the most frequently detected psychoactive substance (67% of the total analyzed products). On the other hand, 3-FMC and pentedrone were the main compounds in products 11 and 12, respectively. Also present, in product 12, was an isomeric impurity identified as isopentedrone (1-methylamino-1-phenylpentan-2-one), a by-product of the pentedrone synthesis, in which the amino moiety and keto group changed their position in the molecule. In relation to the adulterant compounds found in these products, caffeine was the most frequently detected substance (67% of the total analyzed products), followed by ethylphenidate (50% of the total analyzed products).

### 2.3. NMR Analysis

In order to complement the FTIR and GC-MS results, NMR analyzes were performed for a correct structural elucidation. The ^1^H and ^13^C NMR spectra of all substances were registered at 400 and 100 MHz, respectively. Signals assignments were based on chemical shifts (δ, ppm) of ^1^H and ^13^C, on the multiplicity patterns of proton resonances depicted by the *J* couplings (Hz), and on data of homonuclear ^1^H-^1^H COSY and heteronuclear ^1^H-^13^C HMBC and HSQC. The NMR experiments and the signals assignments were made for all compounds and presented in Table 3 and Table 4. For product 11, which contains a fluoromethcathinone, a ^19^F NMR spectrum (376.5 MHz) was also recorded and the respective signal assignment was presented in Table 4. For pyrovalerone derivatives, namely MPHP and α-PHP, identified in products 1, and 2, respectively, the NMR spectra (Figures S4–S8, Supporting Information) showed many similarities, but also some differences that allowed unequivocal identification of both compounds. MPHP is a 1,4-disubstituted aromatic compound with a symmetric distribution of protons on the aromatic ring, and for this reason, the ^1^H NMR signal of the aromatic protons exhibits a characteristic splitting pattern constituted by two doublets at 7.96 ppm (H-2′/H-6′) and 7.47 ppm (H-3′/H-5′). The methyl group attached to the *para* position of the benzene ring (H-7′) produced a large singlet at 2.46 ppm, which was also observed by Westphal et al. [44].

In contrast to MPHP, the ^1^H NMR spectrum of α-PHP showed a typical phenyl pattern at 7.65 ppm (*meta*, appears as a 2H triplet), 7.81 ppm (*para*, appears as a 1H triplet), and 8.06 ppm (*ortho*, appears as a 2H doublet), which is consistent with the values reported in the literature [45,46]. The methine proton (H-2) of both pyrovalerone derivatives showed a triplet signal at around 5.3 ppm, and the protons of the alkyl side chain were found between 2.18 ppm (H-3) and 0.76 ppm (H-6). The multiplets of the methylene protons H-1″ and H-4″ of the pyrrolidine-ring appeared as separated signals, centered at 3.07 ppm (^1^H from H-4″), 3.36 ppm (^1^H from H-1″) and 3.74 ppm (two overlapped protons; ^1^H from H-4″ and 1H from H-1″), while the proton signals of H-2″ and H-3″ appeared between 2.01 and 2.30 ppm and were partially overlap with the methylene protons H-3 of the alkyl side chain. Due to their structural similarities, it was also possible to observe several common characteristics in the ^13^C NMR spectra, including the signals for the carbon of the carbonyl group (C-1) around 198 ppm, the chiral carbon C-2 at 70 ppm and the carbons of the aliphatic side chain C-3, C-4, C-5 and C-6 between 13.2 ppm and 30.2 ppm. The carbons C-1″ and C-4″ of pyrrolidine moiety resonate at 52.6 ppm and 55.8 ppm, respectively, while C-2″ and C-3″ signals are partially overlap at 23.3 ppm, in both compounds. The six aromatic carbons appearing as four signals for these pyrovalerone derivatives showed some differences, thus indicating chemical and structural differences between compounds. By the attachment of a substituent group to the benzene ring, the electronic density and consequently the chemical shifts of the carbon atoms will increase or decrease depending on the electronic nature of the substituent and its position in the aromatic ring [47]. In this sense, the presence of a methyl group in the *para* position of the aromatic ring of MPHP leads to a downfield shift of 12 ppm of the C-4′ resonance in comparison with α-PHP. In addition, the carbon of the methyl group C-7′ attached to the aromatic ring produced a signal at around 21 ppm, which is in agreement with the results obtained by Westphal et al. [44]. 

**Figure 7:**
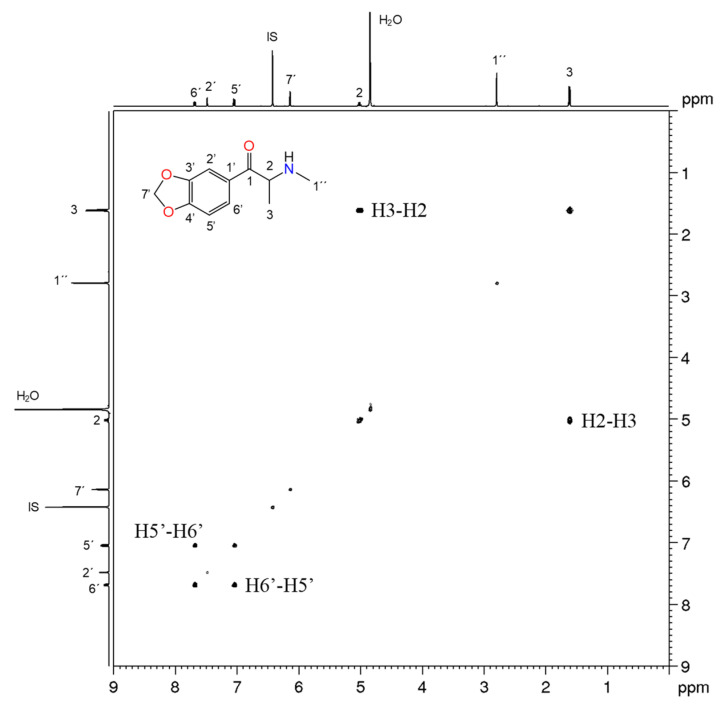
^1^H-^1^H COSY NMR spectrum of methylone found in product 10.

**Figure 8:**
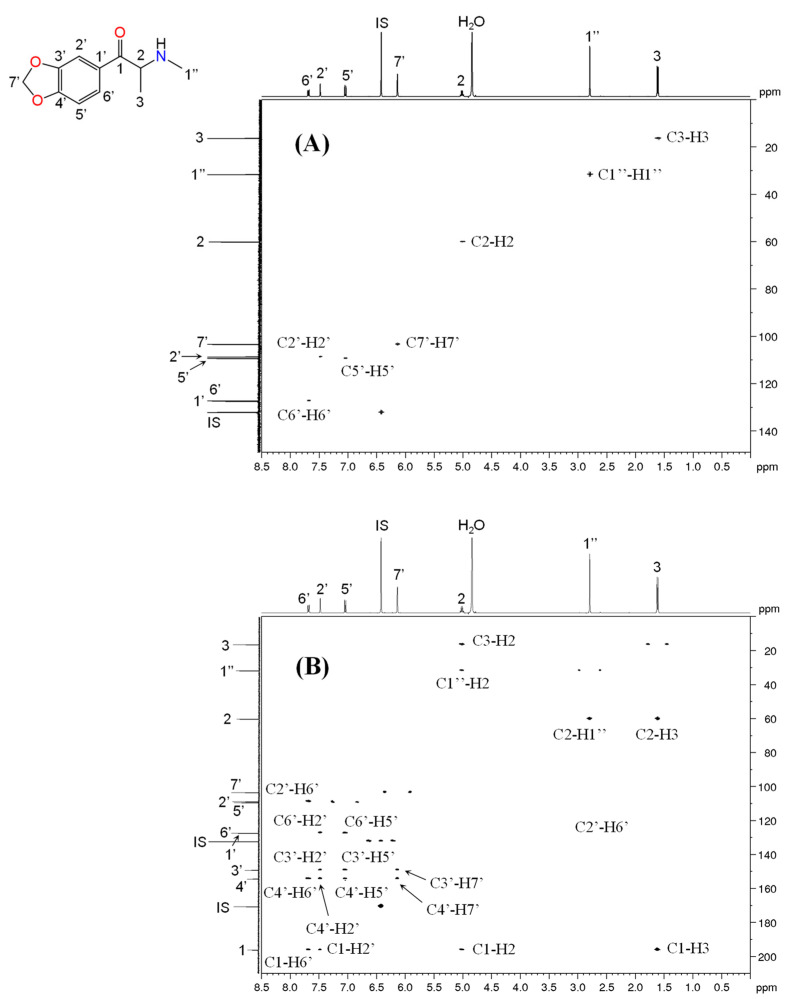
NMR spectra of methylone found in product 10. (**A**) ^1^H-^13^C HSQC and (**B**) ^1^H-^13^C HMBC.

For products 9 and 10, the NMR analysis confirmed the presence of methylone in both samples. Methylone is a 1,3,4-trisubstituted aromatic compound characterized by one double doublet at 7.70 ppm (H-6′) and two doublets at 7.47 (H-2′) and 7.04 ppm (H-5′) on ^1^H NMR spectrum. The methylenedioxy group attached to aromatic ring was identified by the singlet at 6.14 ppm (H-7′), while the methine proton (H-2) located between the carbonyl group and the nitrogen atom were observed at 5.06 ppm (appears as a 1H quartet), which is in agreement with the results reported in the literature [33,48,49]. The singlet at 2.82 ppm integrates 3 protons and belongs to the *N*-methyl group (H-1″), while the doublet at 1.64 ppm corresponds to the terminal methyl group of the alkyl side chain (H-3) that were confirmed by the COSY experiment (Figure 7). In the ^13^C NMR spectrum the signals from the eleven carbon atoms were assigned based on two-dimensional experiments ^1^H-^13^C HSQC and HMBC (Figure 8). The carbon signals corresponding to the phenyl ring were observed at 108.5 (C-2′), 109.1 (C-5′), 127.0 (C-6′), 127.3 (C-1′), 148.9 (C-3′) and 154.1 ppm (C-4′), while the methylenedioxy group (C-7′) produced a signal at around 103 ppm. The carbonyl carbon (C-1) was found at 195.9 ppm, and the methine carbon (C-2) and the two methyl groups C-1″ and C-3 appeared at 59.9, 31.5 and 16.3 ppm, respectively.

Analytical data for products 3–7 revealed the presence of methedrone together with other cathinones, namely *N*-ethylcathinone and buphedrone. Methedrone is a methcathinone derivative only differing by the presence of a methoxy group in the *para* position of the aromatic ring. Hydrogens belonging to the methoxy group were identified in the ^1^H NMR spectrum as a singlet at 3.48 ppm, while the proton attached to chiral carbon (H-2) was identified as a quartet at 5.11 ppm, which are in accordance with the results obtained by Zancajo et al. [50]. An apparent triplet at 8.04 ppm (due to overlap with buphedrone and *N*-ethylcathinone signals) and a doublet at 7.14 ppm, with coupling constants of 8.84 Hz and 8.96 Hz, supported substitution in *para* position. The methyl groups H-3 and H-1″ produce a doublet at 1.64 ppm, and a singlet at 31.6 ppm, respectively.

*N*-Ethylcathinone and buphedrone have a close similar chemical structure, and for this reason, the protons corresponding to the aromatic ring were found overlapped. The protons in the *ortho* position (H-2′ and H-6′) appeared as a doublet at 8.06 ppm with a coupling constant of 8.20 Hz, while *meta* (H-3′ and H-5′) and *para* (H-4′) protons appear as two apparent triplets at 7.65 ppm and 7.81 ppm with coupling constants of 7.62 Hz and 7.44 Hz, respectively. As achieved by Zancajo et al. [50], both compounds showed the signal corresponding to the protons from chiral carbon atoms (H-2) overlapped and appeared as a multiplet at around 5.19 ppm. The *N*-ethyl side chain of *N*-ethylcathinone yielded two multiplets for methylene protons (H-1″) centered at 3.27 ppm and 3.17 ppm and one triplet for the terminal methyl group H-2″ at 1.39 ppm, which are in agreement with the results obtained by Kuś et al. [51]. For buphedrone the *N*-methyl moiety resonates as a large singlet at 2.82 ppm, and the alkyl side chain presents a multiplet centered at 2.14 ppm for the methylene protons H-3 and a triplet at 0.89 ppm for the terminal methyl group (H-4).

In relation to product 12, the NMR data supported the previous findings of GC-MS and confirmed the presence of pentedrone. As expected, the ^1^H NMR results obtained for this SCat was very similar to the ^1^H NMR results for buphedrone, since both substances have similar chemical structures, differing only in the length of the alkyl chain. Pentedrone is constituted by only one more methylene group than buphedrone. The methinic proton of pentedrone was observed at 5.19 ppm and the triplet is more defined than that verified for buphedrone. The two multiplets observed at 1.37 and 1.23 ppm correspond to the diastereotopic protons H-4, while the methylene protons H-3 appeared at 2.04 ppm as a multiplet. The protons corresponding to the terminal methyl group H-5 remained relatively unchanged at around 0.86 ppm. Maheux and Copeland [29] obtained similar results during the chemical characterization of pentedrone and buphedrone in their study.

For product 11, the NMR analysis confirmed the presence of 3-FMC. The observed ^1^H signals, relative to aliphatic portion of the 3-FMC, are similar to those observed for methedrone and methylone. However, the signals pattern observed in the aromatic region was distinct from these previous ones. The two doublets observed at 7.86 ppm and 7.78 ppm corresponded to the protons at *ortho* position H-2′ and H-6′, respectively, while the multiplet centred at 7.65 ppm and the double triplet at 7.53 ppm belonged to the protons in the *meta* (H-5′) and *para* (H-4′) positions. Moreover, the doublet splitting of the carbon atom signals at position 1′ to 6′ and the spin-spin coupling constants (^1^*J*_C3′-F_ = 244.8 Hz, ^2^*J*_C2′-F_ = 23.0 Hz, ^2^*J*_C4′-F_ = 21.4 Hz, ^3^*J*_C5′-F_ = 7.94 HZ, ^3^*J*_C1′-F_ = 6.75 Hz, ^4^*J*_C6′-F_ = 2.89 Hz) were characteristic for fluorine–carbon interactions, which supported the assignment of the 3′-position for the fluorine atom [52]. On the other hand, the ^19^F NMR chemical shift value for the 3-FMC was −114.3 ppm, which is close related with the reported in the literature [52].

**Figure 9:**
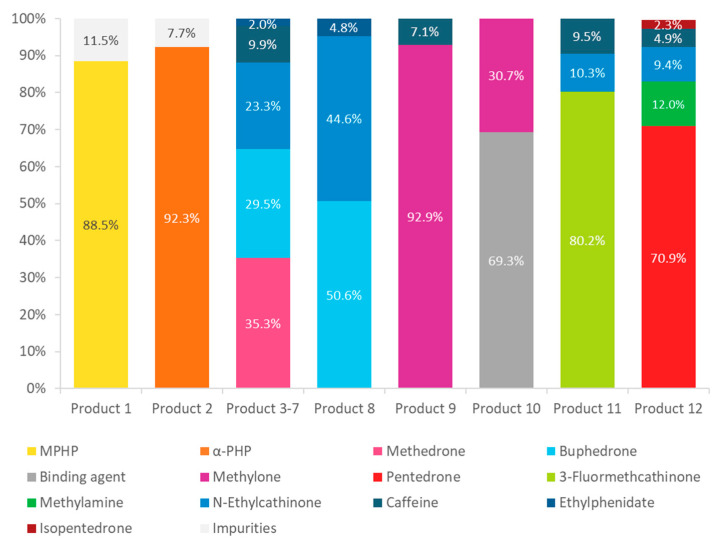
Relative proportions of active substances detected in 12 seized products.

Regarding to the purity of these products, Figure 9 shows the mass fraction (% *m/m*) of each compound found in the seized products. Some of the analyzed products only present one active principle (e.g., product 1, 2 and 10), while most of them are mixtures of psychoactive substances with a high number of constituents, as for example products 3–7 (“Bloom”) or product 12 (“Kick”).

As verified by GC-MS, caffeine and ethylphenidate were the main adulterants found in these samples. The respective assignments of ^1^H and ^13^C signals are described in Table S1 (Supplementary Information). The presence of isopentedrone in product 12 was also confirmed by NMR, being this the minor compound found in this product (2.3%). Additionally, in the same product, NMR analysis allowed the identification of methylamine, which is commonly used in the synthesis of *N*-methyl cathinones and is not detected in the GC-MS analysis due to its volatility [5]. The presence of methylamine in these products was not surprising, since it was previously reported in other works [5,52].

For product 10, only 31% of the analyzed tablets contained methylone in their composition. In fact, during the preparation of this sample for GC-MS and NMR analysis, an insoluble material was observed and was removed from the solution through filtration, and then analyzed by ATR-FTIR. The results are present in Figure S9 (Supporting Information) and the substance was identified as microcrystalline cellulose, which probably was used as a binding agent for direct tablet compression or even added to increase the amount of substance (bulking agent) [53].

## 3. Materials and Methods

### 3.1. Reagents

All used chemicals were of analytical grade. Methanol was obtained from Fisher Chemicals (Loures, Portugal), while ethyl acetate was provided by Riedel-de Haën (Seelze, Germany). Trifluoroacetic anhydride (TFAA ≥ 99.0%), maleic acid (99.0%), trifluoroacetic acid (TFA, 99.0%) and deuterium oxide (99.9%) were obtained from Sigma-Aldrich (St. Louis, MO, USA), while pure caffeine was purchased from Merck (Darmstadt, Germany).

### 3.2. Samples

Twelve seized products suspected to contain illicit substances were provided by the Forensic Science Laboratory of Portuguese Criminal Police, under a special authorization. The products were presented in different forms, including powders, crystals and tablets, packed in small plastic bags with striking designs or, occasionally, in transparent plastic bags. Some of the products were labelled as “plant feeders” with the indication “not for human consumption”. Table 1 shows the list of provided substances, with the respective information about their composition indicated on the product label.

### 3.3. ATR-FTIR Analysis

A PerkinElmer^®^ Spectrum Two FTIR Spectrometer (Waltham, MA, USA) equipped with a DuraSamplIR™ diamond ATR unit (Smiths Detection, London, UK) was used for infrared spectroscopy analysis. Approximately 20 mg of powder sample was placed on the small ATR crystal area, and the infrared spectra were collected in the range from 4000 to 600 cm^−1^, with 32 scans at 4 cm^−1^ resolution. Spectra were obtained in triplicate and a PerkinElmer Spectrum IR software, version 10.6.0, was used for processing and visualizing the spectra.

### 3.4. GC-MS Analysis

For GC-MS analysis, each sample was homogenized and dissolved in methanol to a final concentration of 1 mg mL^−1^. Before the GC-MS analysis, each solution was filtered through 0.22 μm polytetrafluoroethylene (PTFE) membrane filters (Millipore, Milford, MA, USA) and 2 µL of sample were directly injected into the GC-MS. Derivatization with TFAA was also performed according to the method developed by Araújo et al. [5] with some modifications. Each sample was prepared to a final concentration of 100 μg mL^−1^ by dilution of the initial methanolic solution (1 mg mL^−1^) and was evaporated to dryness under nitrogen flow. Then 100 μL of TFAA/ethyl acetate (1:1, *v/v*) was added to the dried residue and the incubation was performed at 70 °C for 30 min. After cooling to room temperature, the solvent was evaporated to dryness under a nitrogen stream, and the residues were reconstituted in 100 μL of ethyl acetate. A 2 μL aliquot of the resulting solution was injected into the GC-MS system.

GC-MS analysis was performed with an Agilent 6890N GC system (Palo Alto, CA, USA) equipped with a 5975 Mass Selective Detector (Agilent Technologies, Palo Alto, CA, USA). The samples were separated using an Agilent J&W HP-5 capillary column (30 m × 0.32 mm ID, 0.25 μm film thickness), and helium (Air Liquid, Alges, Portugal) was used as the carrier gas, at a constant flow of 1.3 mL min^−1^. The injections were performed in split mode, with a ratio of 40:1. The injector port was heated to 250 °C and 2 µL of sample was injected in the GC system. The initial column temperature was set to 60 °C for 4 min, followed by a temperature ramp of 15 °C min^−1^ to 150 °C, held for 5 min, and 20 °C min^−1^ to 290 °C held for 10 min. The mass spectrometer was operated in electron impact ionization (EI) mode at 70 eV. For the MS system, the temperatures of the transfer line, quadrupole and ionization source were 250, 150, and 230 °C, respectively. The ionization was maintained off during the first 4 min, to avoid solvent overloading. The mass spectra were recorded in range of 40–500 *m*/*z* and acquisition was made in Full Scan mode, with a scan rate of 6 scans/s. All mass spectra were compared with NIST 14 MS library and SWGDRUG MS library version 3.4.

### 3.5. NMR Analysis

For NMR analysis, 10 mg of sample was dissolved in 500 μL of deuterium oxide (D_2_O) containing maleic acid as the internal standard (5 mg mL^−1^). The resulting mixture was transferred to a 5 mm NMR tube and the NMR spectra were acquired in a Brucker Avance II 400 MHz Spectrometer (Bruker BioSpin GmbH, Rheinstetten, Germany), operating at 400.13 MHz for ^1^H NMR and 100.61 MHz for ^13^C NMR. Chemical shifts (δ) were expressed as parts per million (ppm) and referenced to the signal of maleic acid (δH = 6.42, δC = 132.16). Coupling constants (*J*) were reported in units of Hertz (Hz). Structure elucidation by respective assignment of the carbon and proton signals was based on the analysis of NMR spectra obtained by 1D (^1^H and ^13^C) and 2D (COSY, HMBC and HSQC) techniques. For product 11, a ^19^F NMR spectrum (376.5 MHz) was also recorded, and the chemical shifts were referenced to the TFA resonance at −78 ppm.

For NMR purity assessment, the ^1^H signal integration for each compound was calculated calibrating for 100 the area of maleic acid resonance peak. The purity of the products was assessed calculating the mass fraction (% *m/m*) of each compound in the seized products, using the following equation,

(1) $$\%compound\;=\;\frac{I_{X}}{I_{IS}}\;\times \;\frac{N_{IS}}{N_{X}}\;\times \;\frac{M_{X}}{M_{IS}}\;\times \;\frac{m_{IS}}{m_{sample}}\;\times \;P_{IS}$$

where $I_{X}$ and $I_{IS}$ are the integrated area of the compound of interest (*X*) and the internal standard (*IS*), $N_{X}$ and $N_{IS}$ are the numbers of protons generating the selected signals for integration, $M_{X}$ and $M_{IS}$ are the molecular weights in g mol^−1^, $m_{sample}$ and $m_{IS}$ are the masses of the sample and the internal standard, respectively, and $P_{IS}$ is the purity of maleic acid.

## 4. Conclusions

The present study describes the identification of 8 SCat as the key constituents found in 12 seized products. In general, the products showed different compositions between them, although very similar for the products labelled with the same name. Among the identified SCat, six of them, methylone, methedrone, *N*-ethylcathinone, buphedrone, pentedrone and 3-FMC, belong to the “classic cathinone group”, analogues of amphetamines, being considered as the first generation of SCat. Pyrovalerone derivatives, a particular class of SCat, characterized by the presence of a pyrrolidine ring in their chemical structures, were also identified in two distinct products with a high degree of purity. MPHP was found in product 1 with a purity of 89%, while α-PHP was found in product 2 with a purity of 92%. Among the adulterants, caffeine, an active ingredient commonly found in illicit drugs, was the most frequently detected substance (67% of the total analyzed products), followed by ethylphenidate (50% of the total analyzed products). Both substances are probably added to these products, since they are cheap and potentiate the stimulating effect.

In relation to the analytical techniques, the combination of ATR-FTIR, GC-MS and NMR techniques proved to be an effective methodology to unequivocally identify the molecular structure of the SCat present in “plant feeders”, even in the absence of certified standards. While ATR-FTIR provided a quick and presumptive identification of the seized drugs without destroying evidence, the GC-MS allowed the precise identification of the substances, being an excellent tool for confirmatory tests. On the other hand, NMR spectroscopy proved to be of great importance for structural elucidation allowing the positional isomers distinction, and the identification of compounds not detected in the GC-MS, such as methylamine.

## Figures and Tables

**Table 1:** List of provided samples with the information about their chemical composition indicated on the product label.

Sample Number	Product Name	Description	Appearance	Quantity (g)	Composition Indicated on the Label
1	Unknown	n.a. ^1^	White powder	1	n.a. ^1^
2	Flakka	n.a. ^1^	Brownish crystal	5	n.a. ^1^
3	Bloom	Plant feeder	White powder	1	94% Ketones, 5% caffeine, 1% glucose
4	Bloom	Plant feeder	White powder	1	94% Ketones, 5% caffeine, 1% glucose
5	Bloom	Plant feeder	White powder	1	94% Ketones, 5% caffeine, 1% glucose
6	Bloom	Plant feeder	White powder	1	94% Ketones, 5% caffeine, 1% glucose
7	Bloom	Plant feeder	White powder	1	94% Ketones, 5% caffeine, 1% glucose
8	Charlie	Plant feeder	Light yellow powder	1	100% Ketones
9	Bliss	Plant feeder	White powder	1	94% Ketones, 5% caffeine, 1% glucose
10	Bliss	Plant feeder	White tablets	5 tablets	Per Pill: 120 mg lactose, 20 mg magnesium stearate, 100 mg corn starch, 160 mg ketones, 50 mg calcium stearate, 4 mg E142, 6 mg E132, 20 mg E124
11	Blast	Plant feeder	White powder	1	89% Ketones, 10% caffeine, 1% glucose
12	Kick	Plant feeder	White powder	1	94% Ketones, 5% caffeine, 1% glucose

^1^ n.a.: not available.

**Table 2:** Active substances detected by GC-MS, with the respective retention times (RT), molecular formula (MF), molecular weight (MW), base peak and other characteristic ions.

Peak No.	Compound Name	Direct Analysis				Derivatization with TFAA			
		RT (min)	MF	MW	Ions (*m*/*z*)	RT (min)	MF	MW	Ions (*m*/*z*)
1	MPHP ^1^	18.654	C_17_H_25_NO	259	**140**, 91, 119, 41	-	-	-	-
2	α-PHP ^1^	17.950	C_17_H_25_NO	245	**140**, 77, 96, 105	-	-	-	-
3	*N*-Ethylcathinone	10.658	C_11_H_15_NO	177	**72**, 44, 77, 105	12.173	C_13_H_14_F_3_NO_2_	273	**168**, 105, 140, 77
4	Buphedrone	10.785	C_11_H_15_NO	177	**72**, 77, 44, 105	11.813	C_13_H_14_F_3_NO_2_	273	**168**, 105, 77, 110
5	Methedrone	14.820	C_11_H_15_NO_2_	193	**58**, 135, 77, 92	16.096	C_13_H_14_F_3_NO_3_	289	**135**, 77, 154, 92
6	Ethylphenidate	17.357	C_14_H_19_NO_2_	233	**84**, 91, 56, 164	18.538	C_17_H_20_F_3_NO_3_	343	**180**, 164, 67, 55
7	Caffeine ^1^	17.775	C_8_H_10_N_4_O_2_	194	**194**, 109, 67, 55	-	-	-	-
8	Methylone	16.314	C_11_H_13_NO_3_	207	**58**, 149, 65, 121	17.239	C_13_H_12_F_3_NO_4_	303	**149**, 154, 121, 110
9	3-FMC	10.007	C_10_H_12_FNO	181	**58**, 95, 75, 123	10.837	C_12_H_11_F_4_NO_2_	277	**154**, 110, 123, 95
10	Isopentedrone	11.516	C_12_H_17_NO	191	**120**, 42, 118, 91	13.239	C_14_H_16_F_3_NO_2_	287	**110**, 216, 182, 140
11	Pentedrone	12.083	C_12_H_17_NO	191	**86**, 44, 77, 105	13.098	C_14_H_16_F_3_NO_2_	287	**182**, 140, 105, 77

^1^ Derivatization did not occur; Bold numbers: base peak ion.

**Table 3:** NMR assignments of SCat constituted by a pyrrolidine ring in the side chain or a methoxy or a methylenodioxy group attached to the aromatic ring.

Position	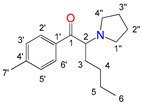		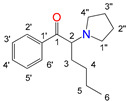		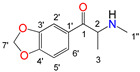		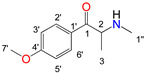	
	MPHP		α-PHP		Methylone		Methedrone	
	^13^C (δ/ppm)	^1^H (δ/ppm, protons, multiplicity ^a^, coupling constants)	^13^C (δ/ppm)	^1^H (δ/ppm, protons, multiplicity ^a^, coupling constants)	^13^C (δ/ppm)	^1^H (δ/ppm, protons, multiplicity ^a^, coupling constants)	^13^C (δ/ppm)	^1^H (δ/ppm, protons, multiplicity ^a^, coupling constants)
1	197.9	-	198.4	-	195.9	-	196.4	-
2	69.9	5.27, 1H, t, *J* = 5.06 Hz	70.0	5.31, 1H, t, *J* = 5.06 Hz	59.9	5.06–5.01, 1H, q, *J* = 7.23 Hz	59.9	5.11–5.05, 1H, q, *J* = 7.21 Hz
3	30.2	2.18–2.01, 2H, m	30.0	2.18–2.01, 2H, m	16.3	1.64, 3H, d, *J* = 7.24 Hz	16.2	1.64, 3H, d, *J* = 7.24 Hz
4	25.8	1.28–1.22, 1H, m 1.15–1.11, 1H, m	25.3	1.28–1.18, 1H, m 1.15–1.11, 1H, m	-	-	-	-
5	22.4	1.28–1.21, 2H, m	22.3	1.28–1.18, 2H, m	-	-	-	-
6	13.3	0.76, 3H, t, *J* = 6.92 Hz	13.2	0.76, 3H, t, *J* = 7.02 Hz	-	-	-	-
1′	131.6	-	134.1	-	127.3	-	125.8	-
2′	129.7	7.96, 2H, d, *J* = 8.16 Hz	129.5	8.06, 2H, d, *J* = 7.52 Hz	108.5	7.47, 1H, d, *J* = 1.64 Hz	132.2	8.04, 2H, at, *J* = 8.84 Hz
3′	130.6	7.47, 2H, d, *J* = 8.08 Hz	129.9	7.65, 2H, t, *J* = 7.82 Hz	148.9	-	115.1	7.14, 2H, d, *J* = 8.96 Hz
4′	148.4	-	136.3	7.81, 1H, t, *J* = 7.46 Hz	154.1	-	165.4	-
5′	130.6	7.47, 2H, d, *J* = 8.08 Hz	129.9	7.65, 2H, t, *J* = 7.82 Hz	109.1	7.04, 1H, d, *J* = 8.28 Hz	115.1	7.14, 2H, d, *J* = 8.96 Hz
6′	129.7	7.96, 2H, d, *J* = 8.16 Hz	129.5	8.06, 2H, d, *J* = 7.52 Hz	127.0	7.70–7.68, 1H, dd, *J* = 8.32, 1.72 Hz	132.2	8.04, 2H, at, *J* = 8.84 Hz
7′	21.6	2.46, 3H, s	-	-	103.2	6.14, 2H, s	56.3	3.48, 3H, s
1″	52.6	3.80–3.69, 1H, m 3.39–3.32, 1H, m	52.6	3.80–3.70, 1H, m 3.40–3.32, 1H, m	31.5	2.82, 3H, s	31.6	2.83, 3H, s
2″	23.3	2.18–2.01, 2H, m	23.3	2.18–2.01, 2H, m	-	-	-	-
3″	23.4	2.30–2.23, 1H, m 2.18–2.01, 1H, m	23.4	2.27–2.21, 1H, m 2.18–2.01, 1H, m	-	-	-	-
4″	55.8	3.80–3.69, 1H, m 3.10–3.04, 1H, m	55.8	3.80–3.70, 1H, m 3.12–3.06, 1H, m	-	-	-	-

^a^ abbreviations: s = singlet, d = doublet, t = triplet, q = quartet, m = multiplet, at = apparent triplet, dd = doublet of doublets. *J* = coupling constant.

**Table 4:** NMR assignments of *N*-ethylcathinone, buphedrone, pentedrone and 3-FMC found in seized materials.

Position	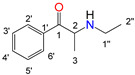		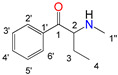		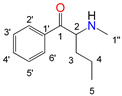		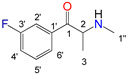		
	*N*-Ethylcathinone		Buphedrone		Pentedrone		3-Fluoromethcathinone		
	^13^C (δ/ppm)	^1^H (δ/ppm, protons, multiplicity ^a^, coupling constants)	^13^C (δ/ppm)	^1^H (δ/ppm, protons, multiplicity ^a^, coupling constants)	^13^C (δ/ppm)	^1^H (δ/ppm, protons, multiplicity ^a^, coupling constants)	^19^F (δ/ppm)	^13^C (δ/ppm)	^1^H (δ/ppm, protons, multiplicity ^a^, coupling constants)
1	198.2	-	197.8	-	197.8	-		196.9, d, *J* = 2.15 Hz	-
2	58.6	5.22–5.16, 1H, m	65.0	5.22–5.16, 1H, m	64.2	5.19, 1H, t, *J* = 5.26 Hz		60.4	5.14–5.09, 1H, q, *J* = 7.28 Hz
3	16.1	1.63, 3H, d, *J* = 7.4 Hz	23.6	2.24–2.05, 2H, m	32.4	2.12–1.95, 2H, m		15.7	1.65, 3H, d, *J* = 7.28 Hz
4	-	-	7.9	0.89, 3H, t, *J* = 7.58 Hz	17.6	1.41–1.32, 1H, m 1.29–1.17, 1H, m		-	-
5	-	-	-	-	13.4	0.86, 3H, t, *J* = 7.28 Hz		-	-
1′	132.9	-	133.7	-	136.0	-		134.9, d, *J* = 6.75 Hz	-
2′	129.5	8.06, 2H, d, *J* = 8.20 Hz	129.4	8.06, 2H, d, *J* = 8.20 Hz	129.4	8.05, 2H, d, *J* = 7.40 Hz		115.9, d, *J* = 23.0 Hz	7.86, 1H, d, *J* = 7.76
3′	129.9	7.65, 2H, at, *J* = 7.62 Hz	129.9	7.65, 2H, at, *J* = 7.62 Hz	129.8	7.64, 2H, t, *J* = 7.82 Hz	−114.3	162.0, d, *J* = 244.8 Hz	-
4′	136.0	7.81, 1H, at, *J* = 7.44 Hz	136.1	7.81, 1H, at *J* = 7.44 Hz	133.6	7.80, 1H, t, *J* = 7.48 Hz		122.8, d, *J* = 21.4 Hz	7.53, 1H, dt, *J* = 8.30, 2.13Hz
5′	129.9	7.65, 2H, at, *J* = 7.62 Hz	129.9	7.65, 2H, at, *J* = 7.62 Hz	129.8	7.64, 2H, t, *J* = 7.82 Hz		131.8, d, *J* = 7.94 Hz	7.68–7.62, 1H, m
6′	129.5	8.06, 2H, d, *J* = 8.20 Hz	129.4	8.06, 2H, d, *J* = 8.20 Hz	129.4	8.05, 2H, d, *J* = 7.40 Hz		125.6, d, *J* = 2.89 Hz	7.78, 1H, d, *J* = 9.28 Hz
1″	41.9	3.31–3.22, 1H, m 3.22–3.13, 1H, m	32.2	2.82, 3H, s	32.3	2.81, 3H, s		31.5	2.85, 3H, s
2″	11.3	1.39, 3H, t, *J* = 7.32 Hz	-	-	-	-		-	-

^a^ abbreviations: s = singlet, d = doublet, t = triplet, q = quartet, m = multiplet, at = apparent triplet, dt = double triplet, *J* = coupling constant.

## Data Availability

No new data were created or analyzed in this study. Data sharing is not applicable to this article.

## Supplementary Materials

The following are available online at https://www.mdpi.com/2218-1989/11/3/144/s1, Figure S1: FTIR spectra of seized products suspected to contain SCat, Figure S2: Typical GC-MS chromatograms of seized products suspected to contain SCat, Figure S3: Typical GC-MS chromatograms of seized products after derivatization with TFAA, Figure S4: ^1^H NMR spectra of seized products, Figure S5: ^13^C NMR spectra of seized products, Figure S6: ^1^H-^1^H COSY NMR spectra of MPHP and α-PHP found in products 1 and 2, respectively, Figure S7: ^1^H-^13^C HSQC and HMBC NMR spectra of MPHP found in product 1, Figure S8: ^1^H-^13^C HSQC and HMBC NMR spectra of α-PHP found in product 2, Figure S9: FTIR spectra of the insoluble substance found in product 10, Table S1: ^1^H and ^13^C NMR assignments of adulterants found in seized materials.
