# Supplementary material for: Structure Assignment of Seized Products Containing Cathinone Derivatives Using High Resolution Analytical Techniques
Source: Metabolites. 2021 Feb 27;11(3):144. doi: 10.3390/metabo11030144 (PMC7997216; doi:10.3390/metabo11030144)
Supplement: Supplementary file 1 [file metabolites-11-00144-s001.pdf]

Metabolite structure assignment of seized products containing cathinone derivatives through high resolution analytical techniques

João L. Gonçalves, Vera L. Alves, Joselin Aguiar, Maria J. Caldeira, Helena M. Teixeira, José S. Câmara*

*CQM - Centro de Química da Madeira, Universidade da Madeira, Campus Universitário da Penteada, 9020-105 Funchal, Portugal.

Supporting Information

Table of Contents

Figure S1.	FTIR spectra of seized products suspected to contain SCat
Figure S2.	Typical GC-MS chromatograms of seized products suspected to contain SCat
Figure S3.	Typical GC-MS chromatograms of seized products after derivatization with TFAA
Figure S4.	^{1}H NMR spectra of seized products
Figure S5.	^{13}C NMR spectra of seized products
Figure S6.	^{1}H-^{1}H COSY NMR spectra of MPHP and α-PHP found in products 1 and 2, respectively
Figure S7.	^{1}H-^{13}C HSQC and HMBC NMR spectra of MPHP found in product 1
Figure S8.	^{1}H-^{13}C HSQC and HMBC NMR spectra of α-PHP found in product 2
Figure S9.	FTIR spectra of the insoluble substance found in product 10
Table S1.	^{1}H and ^{13}C NMR assignments of adulterants found in seized materials

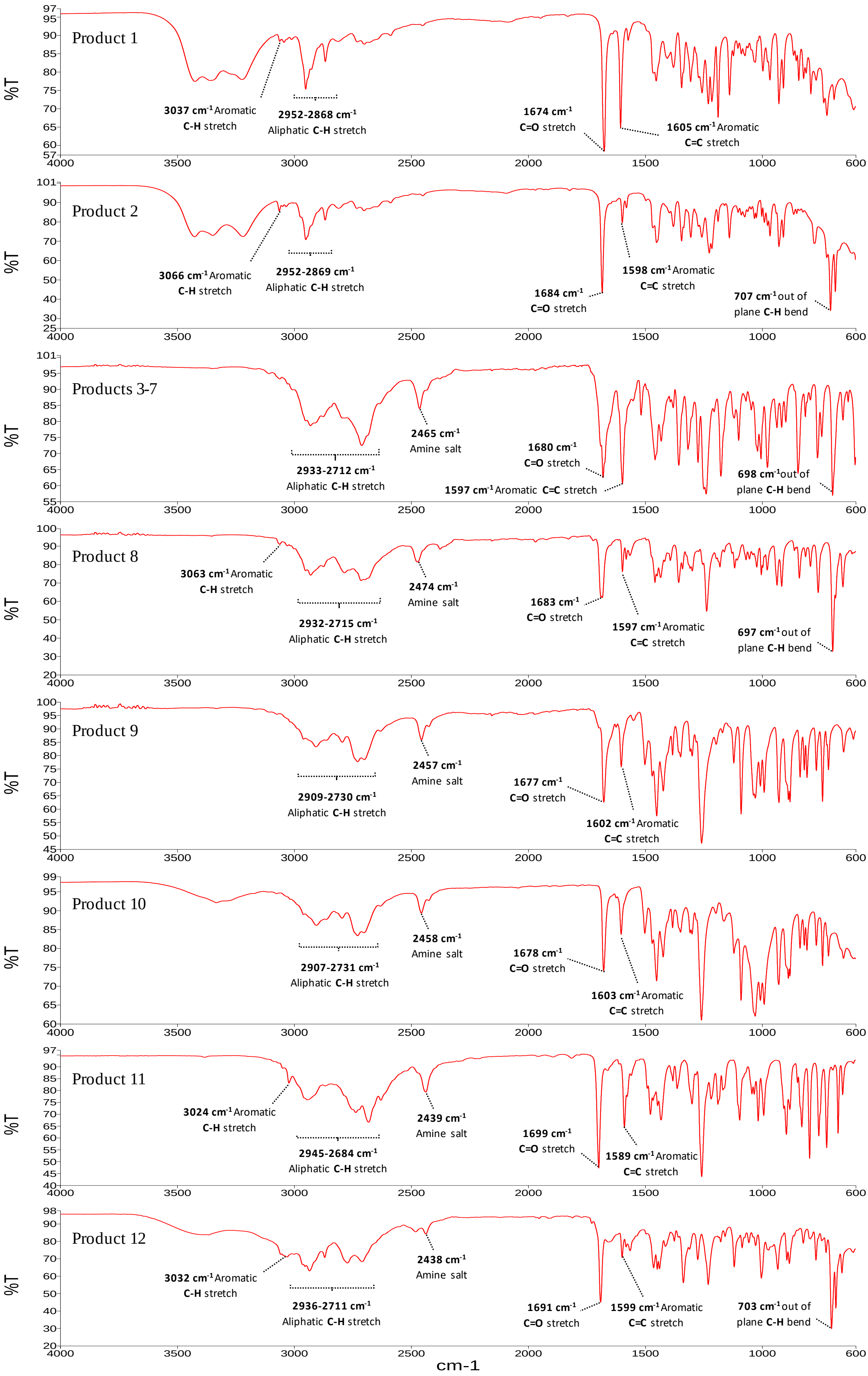

Figure S1. FTIR spectra of seized products suspected to contain SCat. Products 3-7 show similar infrared spectra.

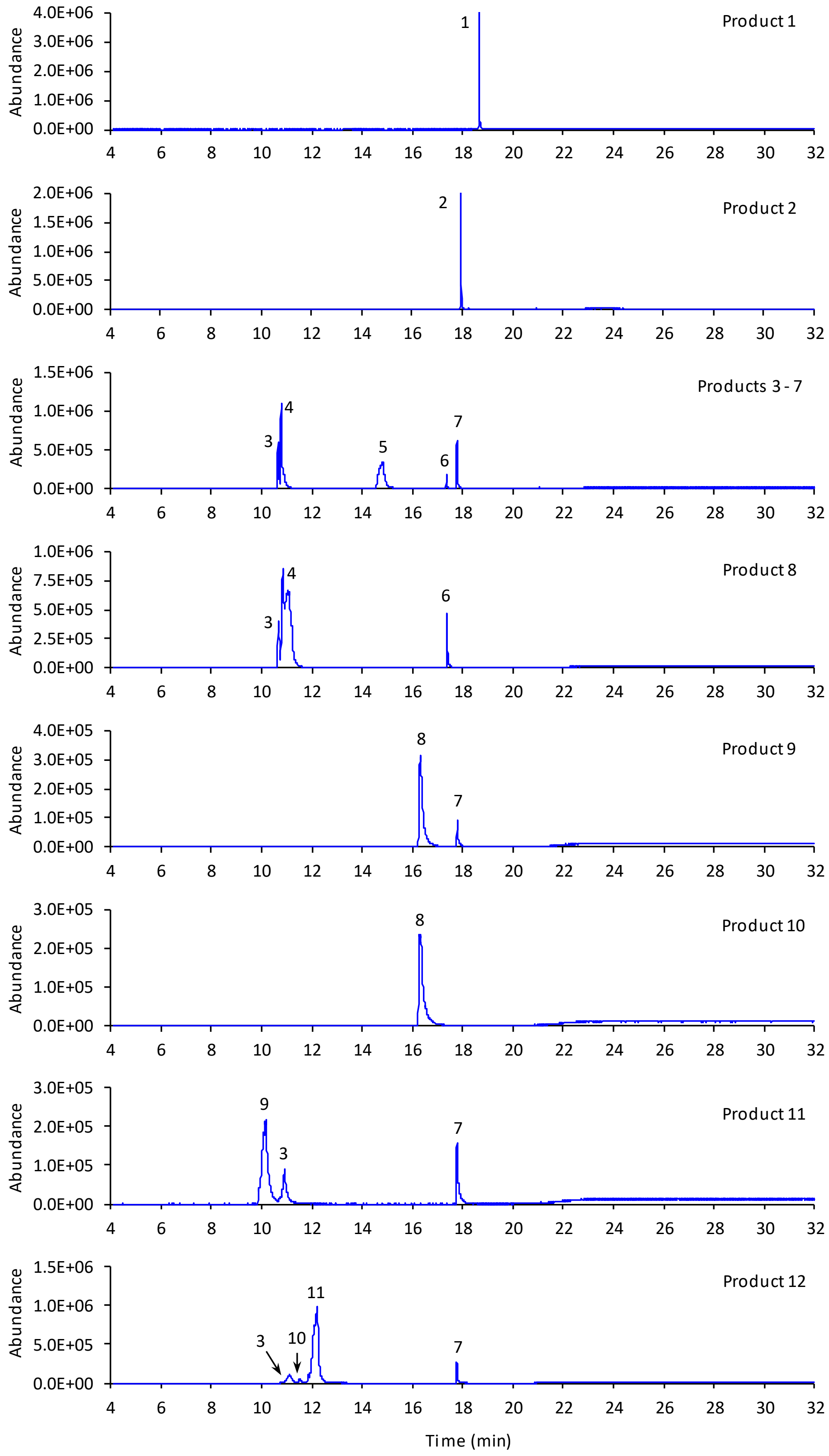

Figure S2. Typical GC-MS chromatograms of seized products suspected to contain SCat. Products 3-7 show similar chromatographic profiles. Peak identification: (1) MPHP, (2) α-PHP, (3) *N*-ethylcathinone, (4) buphedrone, (5) methedrone, (6) ethylphenidate, (7) caffeine, (8) methylone, (9) 3-FMC, (10) Isopentedrone and (11) pentedrone.

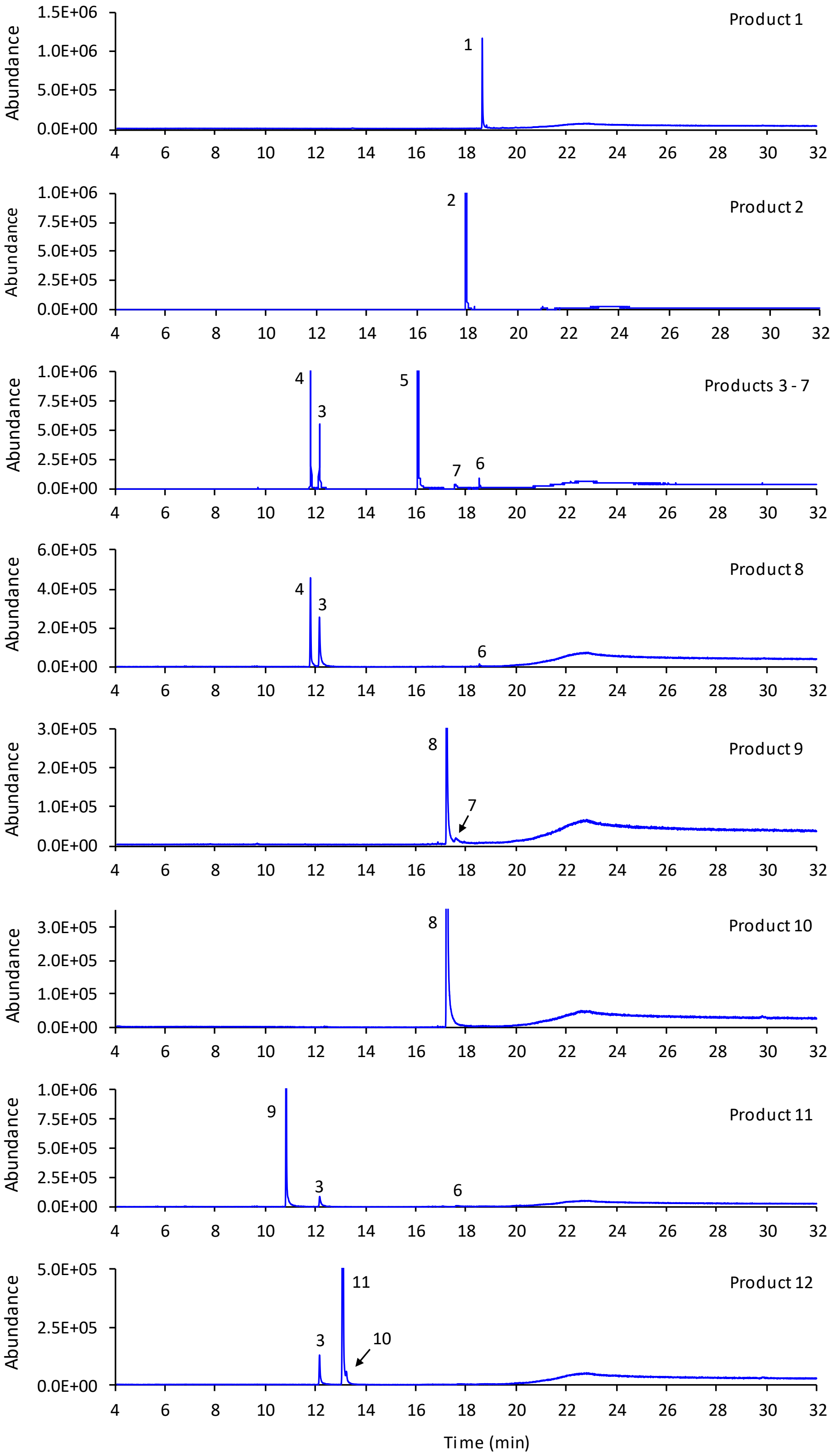

Figure S3. Typical GC-MS chromatograms of seized products after derivatization with TFAA. Products 3-7 show similar chromatographic profiles. Peak identification: (1) MPHP, (2) α-PHP, (3) *N*-ethcathinone-TFA derivative, (4) buphedrone-TFA derivative, (5) methedrone-TFA derivative, (6) ethylphenidate-TFA derivative, (7) caffeine, (8) methylone-TFA derivative, (9) 3-FMC-TFA derivative, (10) Isopentedrone-TFA derivative and (11) pentedrone-TFA derivative.

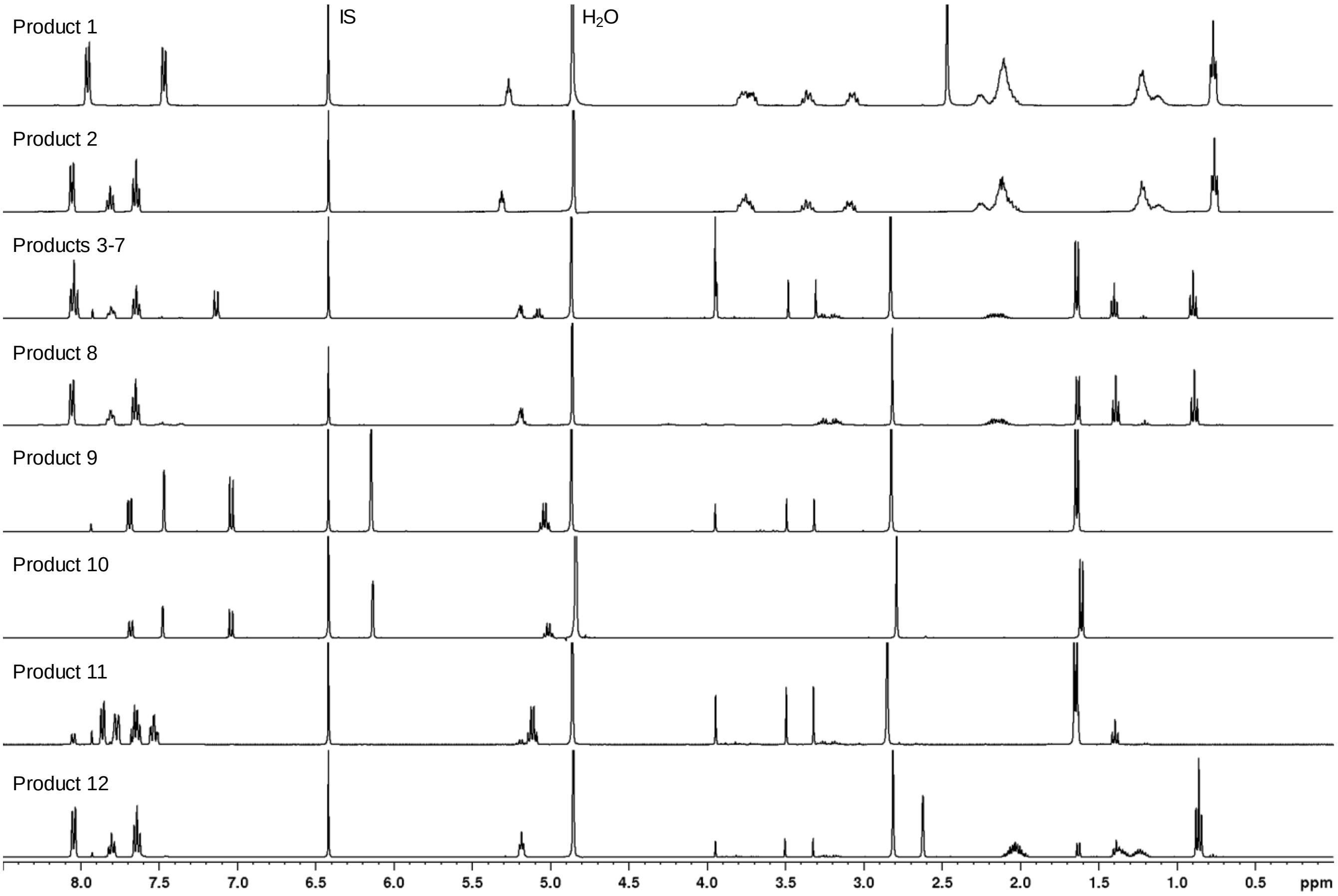

Figure S4. ^{1}H NMR spectra of seized products.

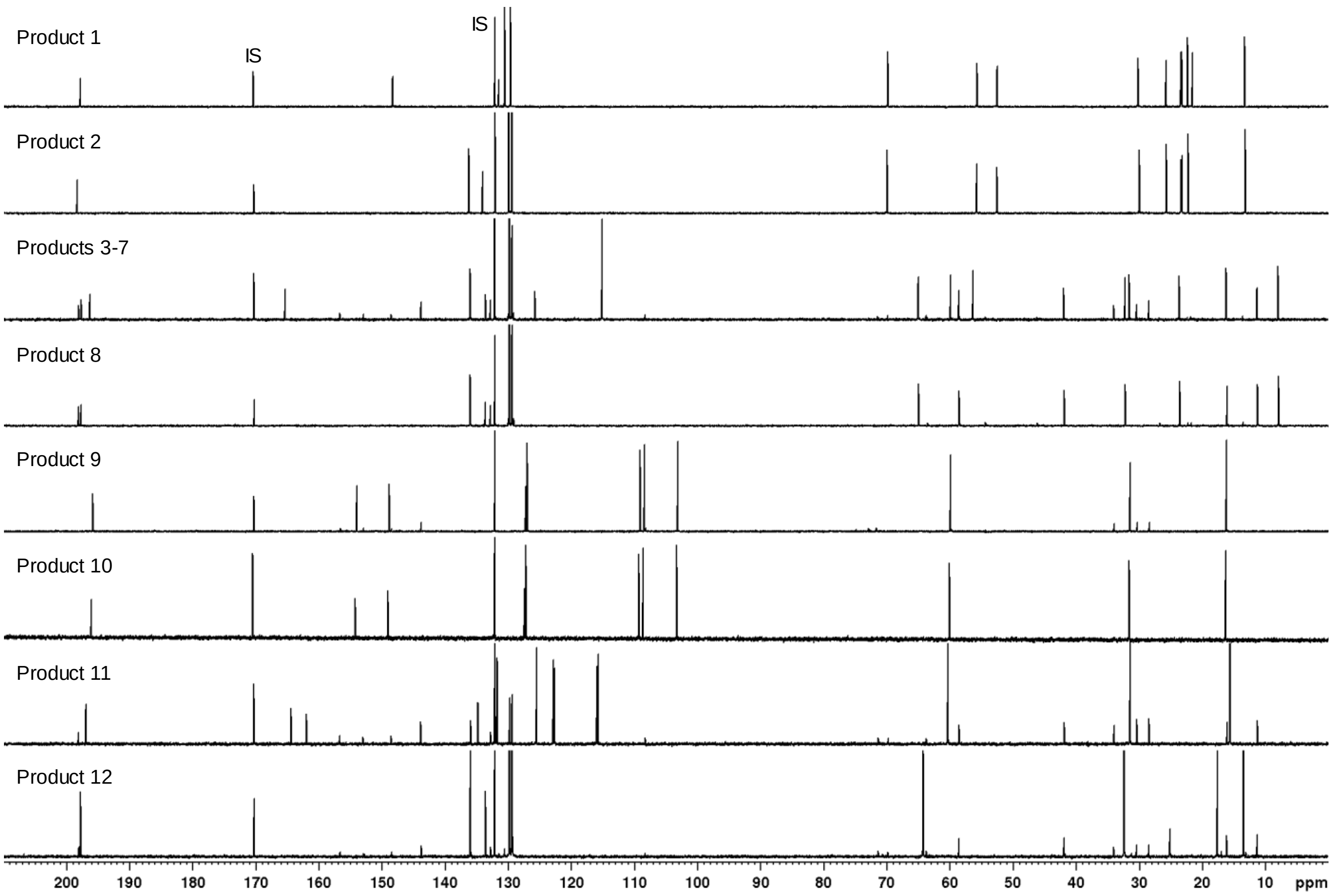

Figure S5. ^{13}C NMR spectra of sezied products.

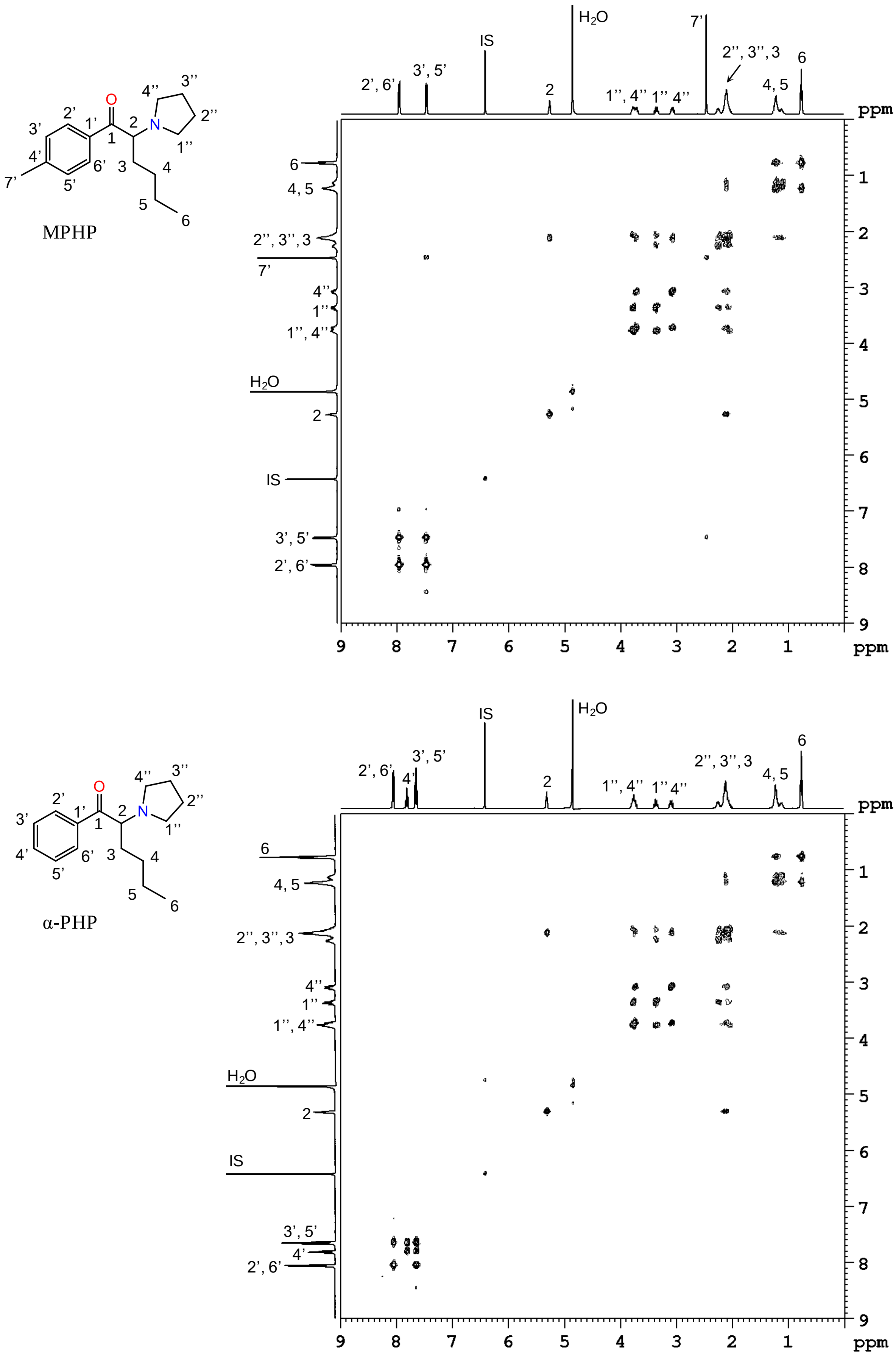

Figure S6. ^{1}H-^{1}H COSY NMR spectra of MPHP and α-PHP found in products 1 and 2, respectively .

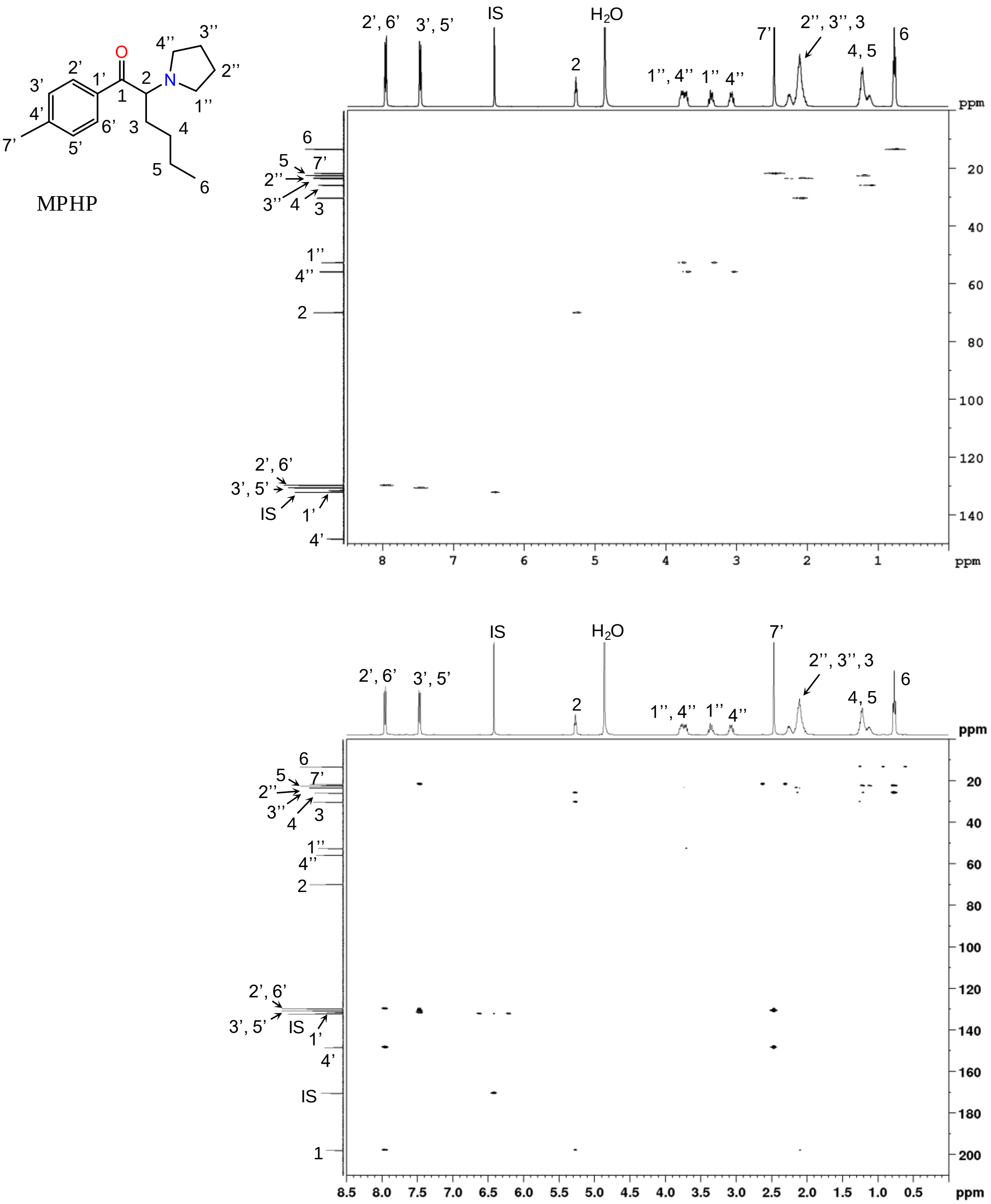

Figure S7. ^{1}H-^{13}C HSQC and HMBC NMR spectra of MPHP found in product 1.

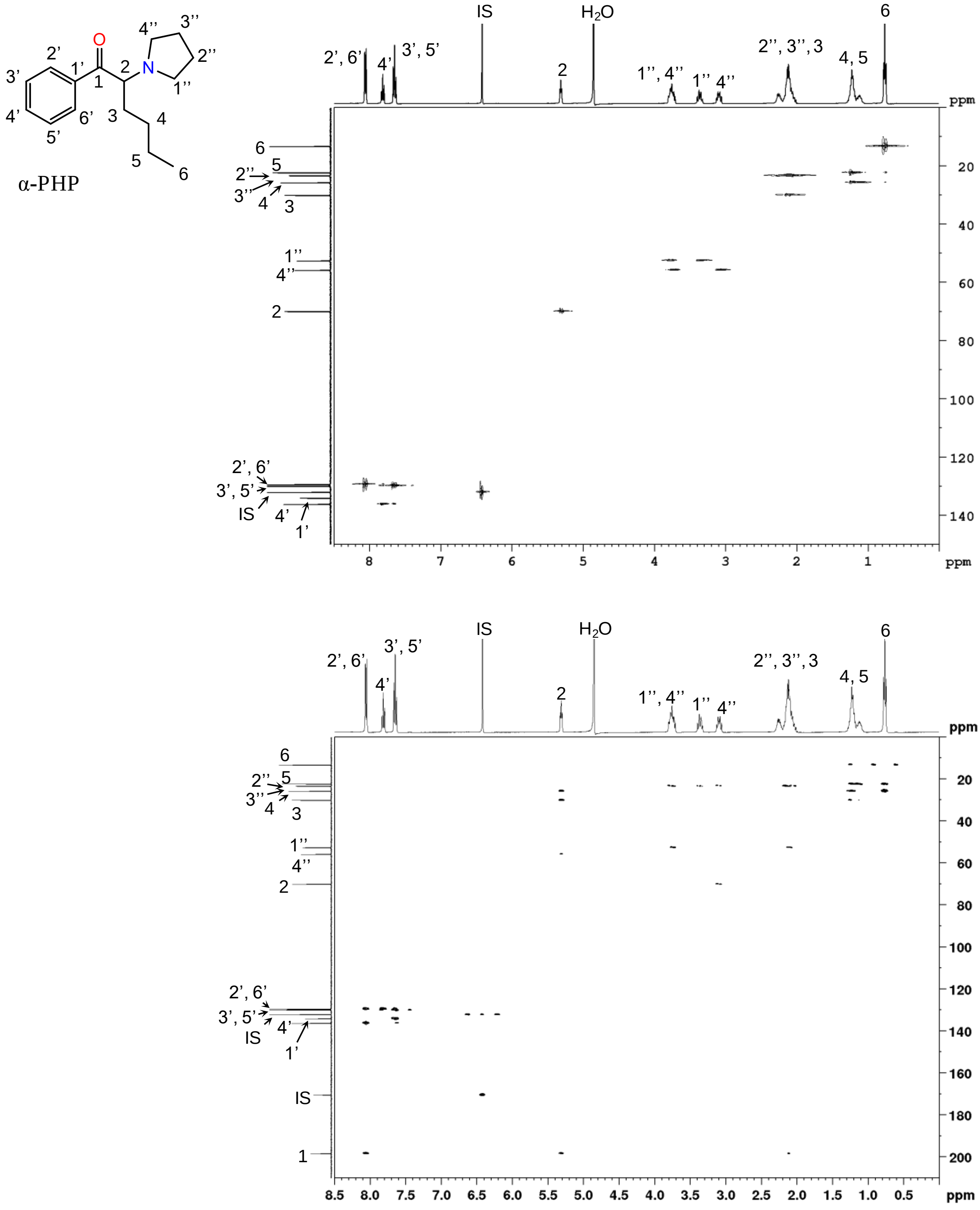

Figure S8. ^{1}H-^{13}C HSQC and HMBC NMR spectra of α-PHP found in product 2.

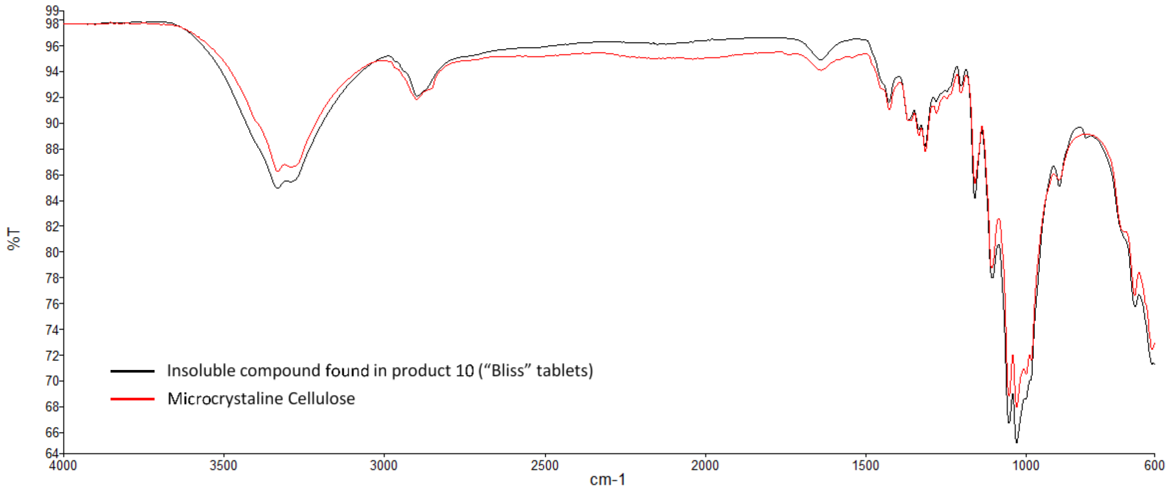

Figure S9. Comparison of the FTIR spectra of the insoluble substance found in product 10 ("Bliss" tablets) with microcristalline cellulose.

From Figure S9, it was possible to notice the following characteristics: the absorbance peaks in the 3400–3300 cm^{-1} regions are attributed to the stretching vibrations of the OH group. The peaks around 2900–2800 cm^{-1} correspond to CH stretching. The band located at 1639 cm^{-1} corresponds to vibration of water molecules adsorbed in microcellulose. The peaks observed in the range of 1420–1430 cm^{-1} were attributed to the symmetric CH_2 bending vibrations, while the absorbance bands at around 1030 cm^{-1} and 896 cm^{-1} were associated with the C-O stretching vibration and the C-H rocking vibration, respectively.

Table S1. ^{1}H and ^{13}C NMR assignments of adulterants found in seized materials.

Position	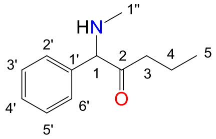<br>Isopentedrone		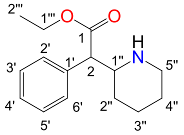<br>Ethylphenidate		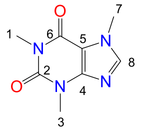<br>Caffeine		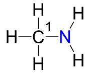<br>Methylamine	
	^{13}C (δ/ppm)	^{1}H (δ/ppm, protons, multiplicity[a], coupling constants)	^{13}C (δ/ppm)	^{1}H (δ/ppm, protons, multiplicity[a], coupling constants)	^{13}C (δ/ppm)	^{1}H (δ/ppm, protons, multiplicity[a], coupling constants)	^{13}C (δ/ppm)	^{1}H (δ/ppm, protons, multiplicity[a], coupling constants)
1	69.9	5.29, 1H, s	173.3	-	28.6	3.26, 3H, s	25.1	2.62, 3H, s
2	206.8	-	54.4	4.02, 1H, d, J = 9.0 Hz	152.8	-	-	-
3	41.7	2.56-5.52, 1H, m<br>2.47-2.38, 1H, m	-	-	30.5	3.43, 3H, s	-	-
4	16.9	1.58-1.49, 3H, m	-	-	148.6	-	-	-
5	13.0	0.70, 3H, t, J = 7.44 Hz	-	-	108.0	-	-	-
6	-	-	-	-	156.3	-	-	-
7	-	-	-	-	34.2	3.91, 3H, s	-	-
8	-	-	-	-	144.0	7.92, 1H, s	-	-
1’	130.6	-	133.9	-	-	-	-	-
2’	129.5	All aromatic signals at 7.46-7.44, 5H, m	129.2	7.36, 2H, ad, J = 7.7 Hz	-	-	-	-
3’	129.6		130.0	7.48, 2H, at, J = 6.2 Hz	-	-	-	-
4’	129.4		129.2	7.44, 1H, at, J = 6.2 Hz	-	-	-	-
5’	129.6		130.0	7.48, 2H, at, J = 6.2 Hz	-	-	-	-
6’	129.5		129.2	7.36, 2H, ad, J = 7.7 Hz	-	-	-	-
1’’	31.2	2.99, 3H, s	58.5	3.86, 2H, at, J = 10.2 Hz	-	-	-	-
2’’	-	-	26.8	1.48-1.45, 1H, m<br>1.68, 1H, m	-	-	-	-
3’’	-	-	21.8	1.84-1.79, 1H, m<br>1.57-1.50, 1H, m	-	-	-	-
4’’	-	-	22.3	1.91, 1H, d, J = 14.2 Hz<br>1.68, 1H, m	-	-	-	-
5’’	-	-	46.2	3.49, 1H, bd, J = 12.9 Hz<br>3.10, 1H, m	-	-	-	-
1’’’	-	-	63.6	4.29-4.20, 2H, m	-	-	-	-
2’’’	-	-	13.6	1.21, 3H, t, J = 7.16 Hz	-	-	-	-

[a]abbreviations: s = singlet, d = doublet, t = triplet, m = multiplet, ad = apparent doublet, at = apparent triplet, bd = broad doublet.
